# *MALAT1* expression is associated with aggressive behavior in indolent B-cell neoplasms

**DOI:** 10.1038/s41598-023-44174-8

**Published:** 2023-10-06

**Authors:** Elena María Fernández-Garnacho, Ferran Nadeu, Silvia Martín, Pablo Mozas, Andrea Rivero, Julio Delgado, Eva Giné, Armando López-Guillermo, Martí Duran-Ferrer, Itziar Salaverria, Cristina López, Sílvia Beà, Santiago Demajo, Pedro Jares, Xose S. Puente, José Ignacio Martín-Subero, Elías Campo, Lluís Hernández

**Affiliations:** 1 Lymphoid Neoplasm Program, Institut d’Investigacions Biomèdiques August Pi I Sunyer (IDIBAPS), Centre Esther Koplowitz (CEK), Rosselló 153, 08036 Barcelona, Spain; 2 Centro de Investigación Biomédica en Red de Cáncer (CIBERONC), Madrid, Spain; 3 Hospital Clínic of Barcelona, Universitat de Barcelona, Barcelona, Spain; 4 University of Oviedo, Oviedo, Spain; 5 Institució Catalana de Recerca i Estudis Avançats (ICREA), Barcelona, Spain

**Keywords:** Cancer, Molecular medicine, Cancer, Cancer, Haematological diseases

## Abstract

*MALAT1* long non-coding RNA has oncogenic roles but has been poorly studied in indolent B-cell neoplasms. Here, *MALAT1* expression was analyzed using RNA-seq, microarrays or qRT-PCR in primary samples from clinico-biological subtypes of chronic lymphocytic leukemia (CLL, n = 266), paired Richter transformation (RT, n = 6) and follicular lymphoma (FL, n = 61). In peripheral blood (PB) CLL samples, high *MALAT1* expression was associated with a significantly shorter time to treatment independently from other known prognostic factors. Coding genes expressed in association with *MALAT1* in CLL were predominantly related to oncogenic pathways stimulated in the lymph node (LN) microenvironment. In RT paired samples, *MALAT1* levels were lower, concordant with their acquired increased independency of external signals. Moreover, *MALAT1* levels in paired PB/LN CLLs were similar, suggesting that the prognostic value of *MALAT1* expression in PB is mirroring expression differences already present in LN. Similarly, high *MALAT1* expression in FL predicted for a shorter progression-free survival, in association with expression pathways promoting FL pathogenesis. In summary, *MALAT1* expression is related to pathophysiology and more aggressive clinical behavior of indolent B-cell neoplasms. Particularly in CLL, its levels could be a surrogate marker of the microenvironment stimulation and may contribute to refine the clinical management of these patients.

## Introduction

Long non-coding RNAs (lncRNAs) regulate the expression of protein-coding genes and are increasingly described as key players in physiological and pathological conditions, most remarkably in cancer^1,2^. One of the lncRNAs most frequently related to oncogenesis is *MALAT1*, which has been implicated in the regulation of key cellular pathways such as MAPK/ERK, PI3K/AKT, WNT/B-catenin, and NF-kB^3^ and, as a consequence, involved in many cancer-associated processes such as cell proliferation, migration, invasion, apoptosis and angiogenesis^4^. There are also several studies supporting *MALAT1* expression as a clinical biomarker mainly associated with a poor prognosis in solid tumors^3^, although in some neoplasms such as diffuse large B-cell lymphoma (DLBCL), colorectal and breast cancer, high levels of *MALAT1* have been linked to a favorable outcome^5–7^.

In B-cell non-Hodgkin lymphomas (B-NHL), we and others have previously shown that lncRNA deregulation is associated with immune cell-related functions and cell proliferation control^8,9^. In the case of *MALAT1,* its expression has been shown to be upregulated in some lymphoid neoplasms such as DLBCL^10^, chronic lymphocytic leukemia (CLL)^11^, and mantle cell lymphoma (MCL)^12^. These results suggest that *MALAT1* expression could be associated with poor prognosis even in indolent B-NHL, but most of these studies include relatively few cases and the possible clinical relevance of *MALAT1* expression in these lymphoid neoplasms is still not well known. In this study, we have performed a detailed characterization of the biological and clinical impact of *MALAT1* deregulation in two types of indolent B cell lymphomas with different underlying pathobiological mechanisms, namely CLL and follicular lymphoma (FL).

## Results

### MALAT1 expression in CLL

**Figure 1:**
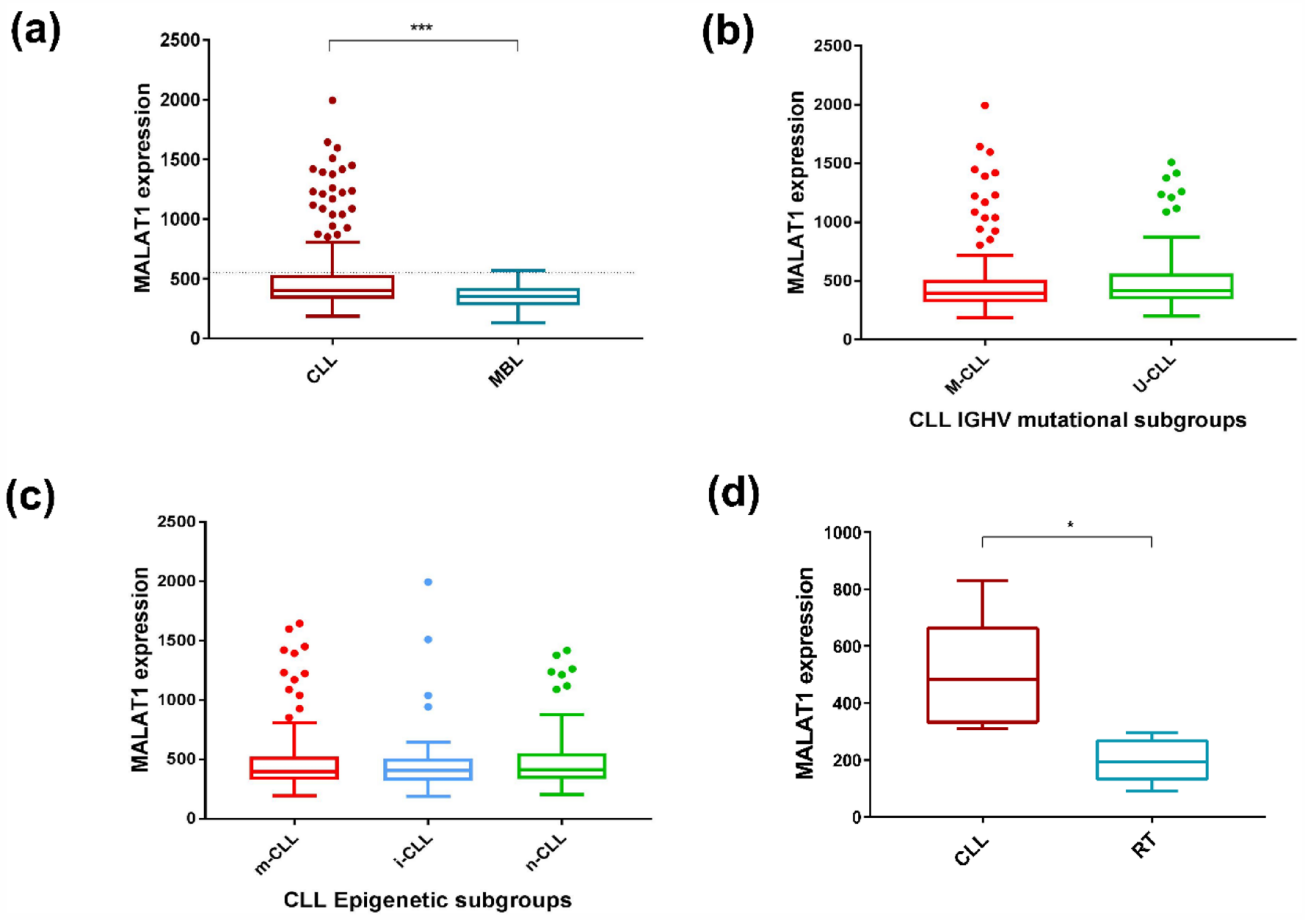
*MALAT1* expression levels in MBL, CLL#1 molecular subgroups, and RT. (**a**) *MALAT1* expression was higher in CLL patients than in MBL (*P* < 0.001). Dotted line indicates the threshold of *MALAT1* that determines differences in outcome. (**b**) No significant differences were observed in *MALAT1* expression between CLL subgroups defined by IGHV mutational status (M-CLL versus U-CLL). (**c**) No significant differences were observed in *MALAT1* expression between CLL subgroups defined by epigenetic subtypes (n-CLL: naïve-like, m-CLL: memory-like and i-CLL: intermediate) (right panel). (**d**) *MALAT1* expression was higher in a series of 6 samples of CLL patients than in their corresponding paired RT samples (*P* < 0.01).

We initially evaluated the expression levels of *MALAT1* in 266 CLL (Cohort CLL#1) and 25 monoclonal B cell lymphocytosis (MBL) (Table 1). *MALAT1* expression was significantly higher in CLL than in MBL (*P* < 0.001) (Fig. 1a). We also stratified *MALAT1* levels according to the IGHV mutational status (mutated -M-CLL- vs un-mutated U-CLL) and epigenetic subtypes (naïve-like -n-CLL, memory-like -m-CLL and intermediate -i-CLL-) and no significant differences were observed among the different groups (Fig. 1b and c). In addition, we analyzed *MALAT1* expression in 6 paired CLL and their clonally related transformation to diffuse large B cell lymphoma (Richter transformation, RT) (CLL#2 series, Table 1). *MALAT1* was significantly downregulated in the RT samples (*P* < 0.01) (Fig. 1d).

**Table 1:** Summary of the studied CLL and FL series.

Pathology	Code	Data source	Tissue	Number of cases	Platform
MBL	MBL	ICGC	PB	25	RNA-seq
CLL	CLL#1	ICGC	PB	266	RNA-seq
	CLL#2	EGAD00001008959	PB	6 CLLvs RT	RNA-seq
	CLL#3	GSE21029	LN/PB/BM	17 LNvsPB & 19 PBvsBM	Microarrays (HGU133Plus2, Affymetrix)
FL	FL#1	Biobank, Hospital Clínic	LN	61	RT-qPCR
	FL#2	GSE107367	LN	23	Microarrays (HGU133Plus2, Affymetrix)

**Figure 2:**
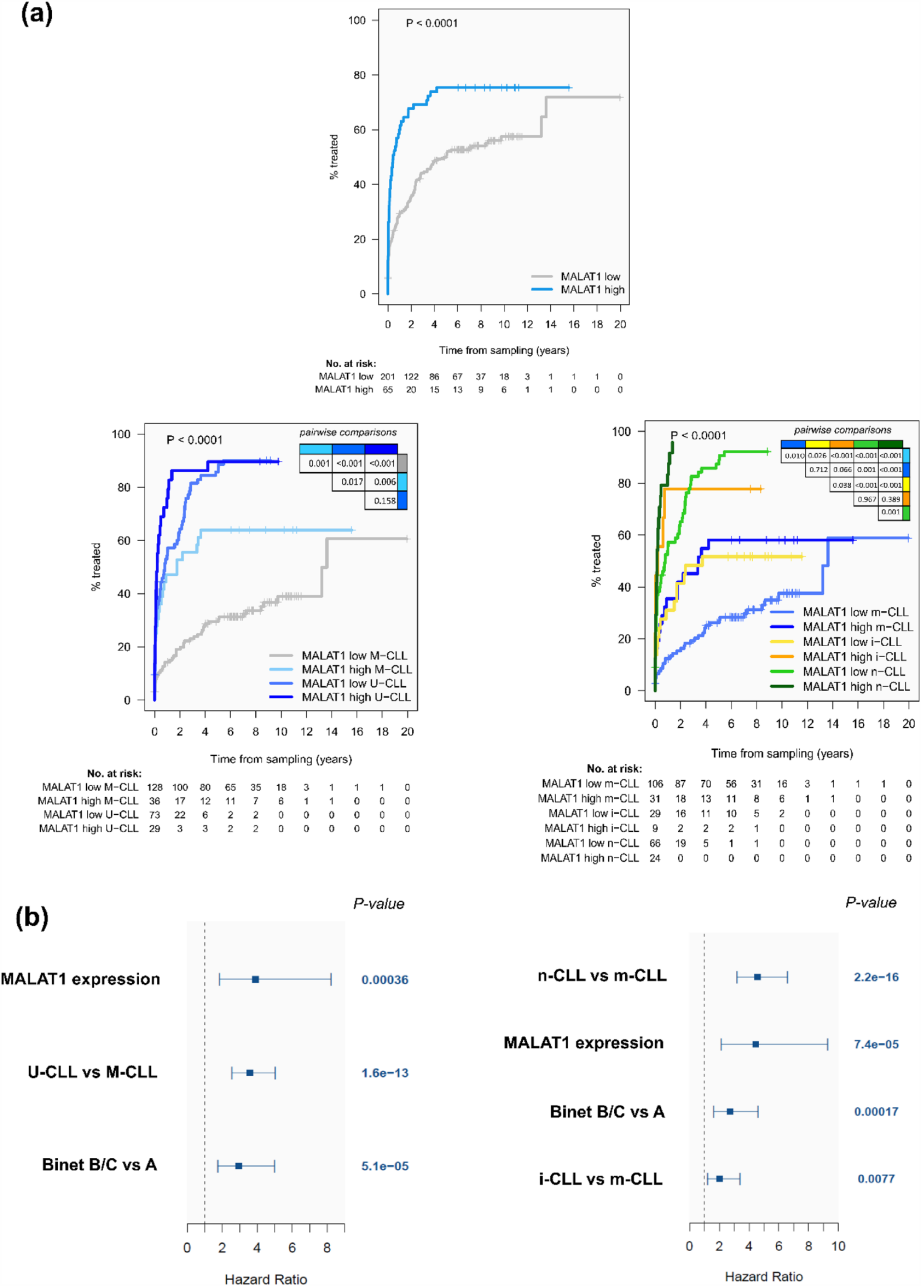
*MALAT1* expression is an independent prognostic factor for time to treatment (TTT) in CLL. (**a**) CLL patients with high *MALAT1* expression showed significantly shorter TTT compared to those with low levels in the global cohort (top panel) and almost every one of the different CLL subgroups related to the IGHV mutational status (bottom left) and epigenetic subtypes (bottom right). (**b**) Plot for multivariate model analyses, where *MALAT1* expression as a continuous variable shows its independent prognostic value considered together with Binet stage and IGHV mutational status (left panel) or epigenetic subtypes (right panel). For epigenetic groups, m-CLL was taken as a reference.

We next evaluated whether *MALAT1* expression was associated with the clinical behavior of the tumor. Using the *maxstat* algorithm, we segregated CLL patients into high and low expression groups (Supplementary Fig. S1a online). Patients with high *MALAT1* expression (N = 65) had a significantly shorter time to treatment (TTT) than the *MALAT1*-low group (N = 201) (*p* < 0.0001) (Fig. 2a). This finding was also confirmed in the subset of patients clinically classified as Binet A (N = 239, 57 with high and 182 low *MALAT1* levels) (*P* = 0.0003) (Supplementary Fig. S2a online). On the contrary, *MALAT1* levels were not related to the overall survival (OS) of the patients in the whole series (Supplementary Fig. S1b and S2b online) or stratifying the patients according to the use (N = 77) or not (N = 60) of rituximab on different chemotherapy regimens (Supplementary Table S1 online) (*P* = 0.618 and *P* = 0.758, respectively, data not shown). The adverse impact of high *MALAT1* expression on TTT was further confirmed in IGHV-mutated CLL and in the three epigenetic CLL subtypes (Fig. 2a). Similar findings were observed when the analyses were restricted to patients with Binet A CLL (Supplementary Fig. S2c,d online). To evaluate the independent prognostic impact of *MALAT1*, we performed multivariate regression models. First, we checked that *MALAT1* as a continuous isolated variable also had significant prognostic value for TTT (HR = 1.32; 95%CI: 1.18–1.48; *p* < 0.0001) but not for OS (HR = 1.05; 95%CI: 0.89–1.24; *p* = 0.561). Next, the multivariate analyses confirmed that *MALAT1* expression also had an independent prognostic value for TTT considering Binet stage and the IGHV mutational status (*P* = 0.0004) and Binet stage and the epigenetic subgroups (*P* < 0.0001) (Fig. 2b). We also evaluated the possible association of *MALAT1* levels with other molecular factors previously shown to have prognostic value in CLL, such as mutations in driver genes, number of chromosomal aberrations^13,14^, IGLV3-21 variant/R110 mutation^15^, or the DNA methylation-based epiCMIT score related to the proliferative history of the tumor cells^16^. *MALAT1* expression levels were not related to any of these variables (Supplementary Fig. S3 and S4 online). Overall, these data indicate that *MALAT1* expression has a prognostic value for TTT in CLL irrespective of other known genetic and epigenetic prognostic parameters.

To determine whether *MALAT1* deregulation could be related to genetic alterations, we analyzed the gene mutational status in the whole-genome sequences of 150 cases from the ICGC Consortium^13^. Only five CLL and one MBL revealed mutations in the *MALAT1* locus and were not related to the expression of the gene (Supplementary Table S2a online). No copy number alterations affecting 11q13, where *MALAT1* is located, were found, whereas only 2 CLL cases had copy number neutral loss of heterozygosity. None of these alterations were related to the expression of the gene (Supplementary Table S2b online). We also consider the possible association of *MALAT1* expression with the methylation status of this gene using previously published data included in the CLL#1 series^16^. The CpGs with methylation status available in the *MALAT1* gene region were mainly hypomethylated, but using clustering analysis we could not observe any association between CpG status and *MALAT1* expression, neither considered as a continuous nor categorical variable in any of the different CLL subtypes (Supplementary Fig. S5 online).

**Figure 3:**
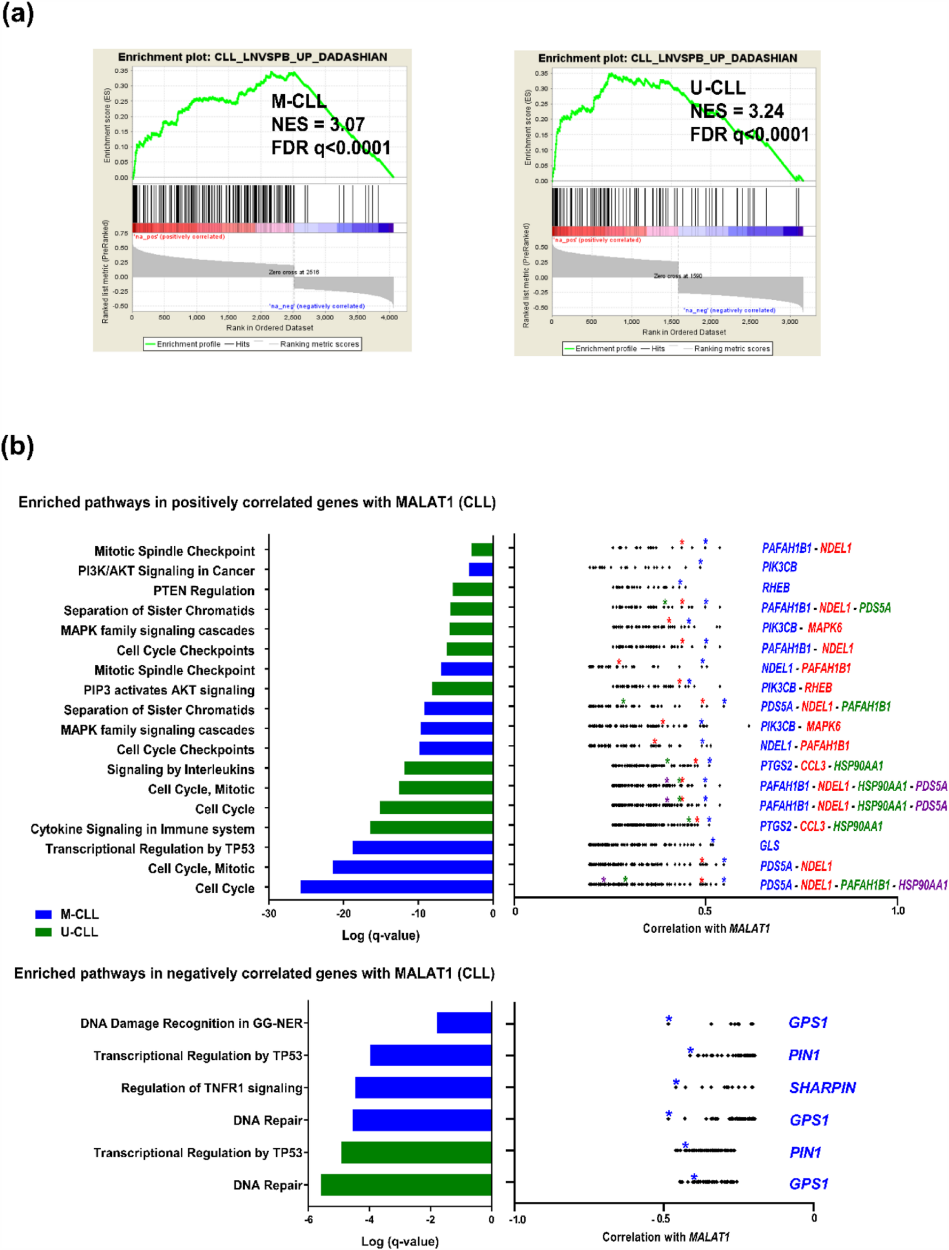
*MALAT1* levels correlate positively with genes enriched in survival and proliferation pathways, including a signature described as upregulated in LN compared to PB CLL samples. (**a**) GSEA analysis shows that a nodal CLL signature (see material and methods) was significantly enriched in coding genes correlated with *MALAT1* expression in PB CLL samples of M-CLL and U-CLL subtypes. (**b**) Summary of most relevant significant pathway enrichments found using Metascape tool (left panels) regarding coding genes positively (top panels) and negatively (bottom panels) correlated with *MALAT1* expression in CLL subtypes defined by IGHV mutational status. Only statistically significant Reactome pathways after multiple comparison correction are shown for compact representation. Correlation values of the core enriched genes of the corresponding pathways are depicted in the right panels with relevant genes marked in colored asterisks according to the degree of correlation (in blue those more highly correlated with *MALAT1*).

To evaluate the possible functional implications of *MALAT1* expression we searched for coding genes that were significantly correlated, either positively or negatively, with *MALAT1* in the different CLL subgroups (M-CLL and U-CLL and epigenetic subtypes) (Supplementary Table S3 online) and we subsequently performed pathway enrichment analyses. The positively correlated genes were significantly enriched in a signature highly expressed in nodal CLL compared to their respective peripheral blood sample. This signature was enriched in both U-CLL and M-CLL (Fig. 3a)^17^. Other pathways identified were related to activation, proliferation, and survival of CLL cells in the lymph node (LN) microenvironment, particularly PI3K/AKT, MAPK, and signaling of several interleukins, among others^18–22^ (Fig. 3b, Supplementary Fig. S6a and Table S4 online). The top core genes enriched in these pathways had been previously related to progression of the cell cycle as *NDEL1*, *PAFAH1B1* and *PDS5A*^23–26^, or involved in cytokine-related pathways such as *CCL3* and *PTGS2* (Fig. 3b and Supplementary Table S5 online). *CCL3* has been critically related to the composition of LN microenvironment promoting CLL survival and proliferation^27^. *PTGS2* is already known to be overexpressed in CLL promoting resistance to apoptosis^28^ and being induced by different factors through STAT3 activation^29,30^. Other noticeable examples were *PIK3CB,* related to PI3K/AKT and MAPK pathways^31^, *RHEB*, a GTPase upstream activator of MTOR that cooperates with *PTEN* haploinsufficiency in MYC-induced murine lymphomas^32^, and *GLS* in the *TP53* transcriptional regulation pathway, overexpressed in many cancers^33^. Moreover, in the MAPK pathway we observed a high correlation with *MAPK6* whose overexpression has been associated with poor survival in several solid tumors^34^.

On the other hand, the analysis of the pathways and their top genes negatively correlated with *MALAT1* showed significantly enrichment of DNA damage and repair pathway (e.g. *GPS1*, a gene described as a suppressor of mitogen-activated signal transduction in addition to the impairing in DNA repair induced by its depletion)^35^, TNFR signaling (*SHARPIN*, a negative regulator of NF-kB via TNFR) and TP53 mediated transcriptional regulation (*PIN1,* an inhibitor of TP53 activation) (Fig. 3b, Supplementary Table S5 and S6 online)^36,37^.

**Figure 4:**
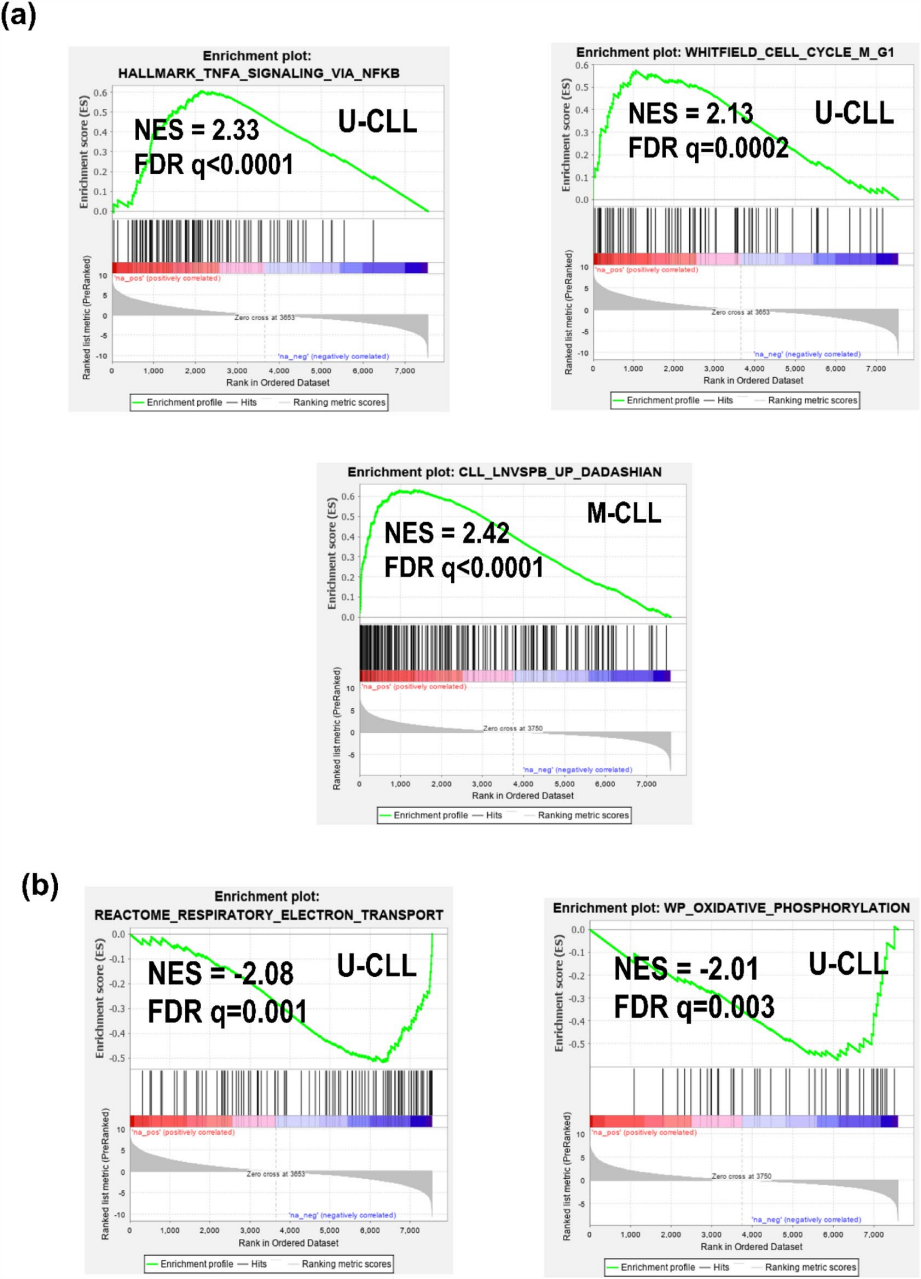
Pathways enriched by GSEA among the genes ranked by their degree of differential expression between high and low *MALAT1* expression groups in CLL IGHV subtypes. (**a**) Positive significantly enriched pathways related to survival, proliferation and a signature of genes described as upregulated in LN vs PB CLL samples. (**b**) Negative significantly enriched pathways related to OXPHOS.

We subsequently analyzed the differentially expressed genes between cases with high and low *MALAT1* expression in U-CLL and M-CLL of the CLL#1 series. A total of 35 (M-CLL) and 65 genes (U-CLL) were found with significant differences in expression (Supplementary Fig. S7 and Table S7 online). These genes include several identified in the previous pathway analysis of correlated genes with *MALAT1* expression and were related to the influence of the microenvironment in the lymph node (e.g. *FOSB*, *DUSP1*, *CD69*, *NR4A2* and *KLF10*) (Supplementary Table S8 online)^17^. To further understand the significance of the differentially expressed genes we performed a GSEA analyses on genes ranked by the degree of differential expression. This analysis confirmed pathways identified in the previous analysis of genes correlated with *MALAT1* expression such as the signature highly expressed in nodal CLL and cell cycle regulation, as well as pathways related to regulation of proliferation and NF-kB activation, among others involved in CLL pathogenesis (Fig. 4a and Supplementary Table S9). We also detected a negative enrichment of OXPHOS-related pathways, particularly for U-CLL (Fig. 4b and Supplementary Table S9 online). Interestingly, this later observation was concordant with the significant *MALAT1* downregulation that we observed in the RT samples, since an increased OXPHOS pathway has been recently identified as characteristic of RT^38^.

Finally, we also analyzed previously published microarray data of paired LN or bone marrow (BM) and PB CLL samples^39^ (CLL#3 series, Table 1), where we found that *MALAT1* levels did not change significantly between LN or BM compared to PB (*P* = 0.097; *P* = 0.698, respectively) (Supplementary Fig. S8 online). All these results suggest that the relationship between *MALAT1* expression and shorter TTT that we have observed in PB samples could represent a surrogate biomarker for the degree of stimulation and proliferation of CLL cells in the LN.

### MALAT1 expression in **FL**

**Figure 5:**
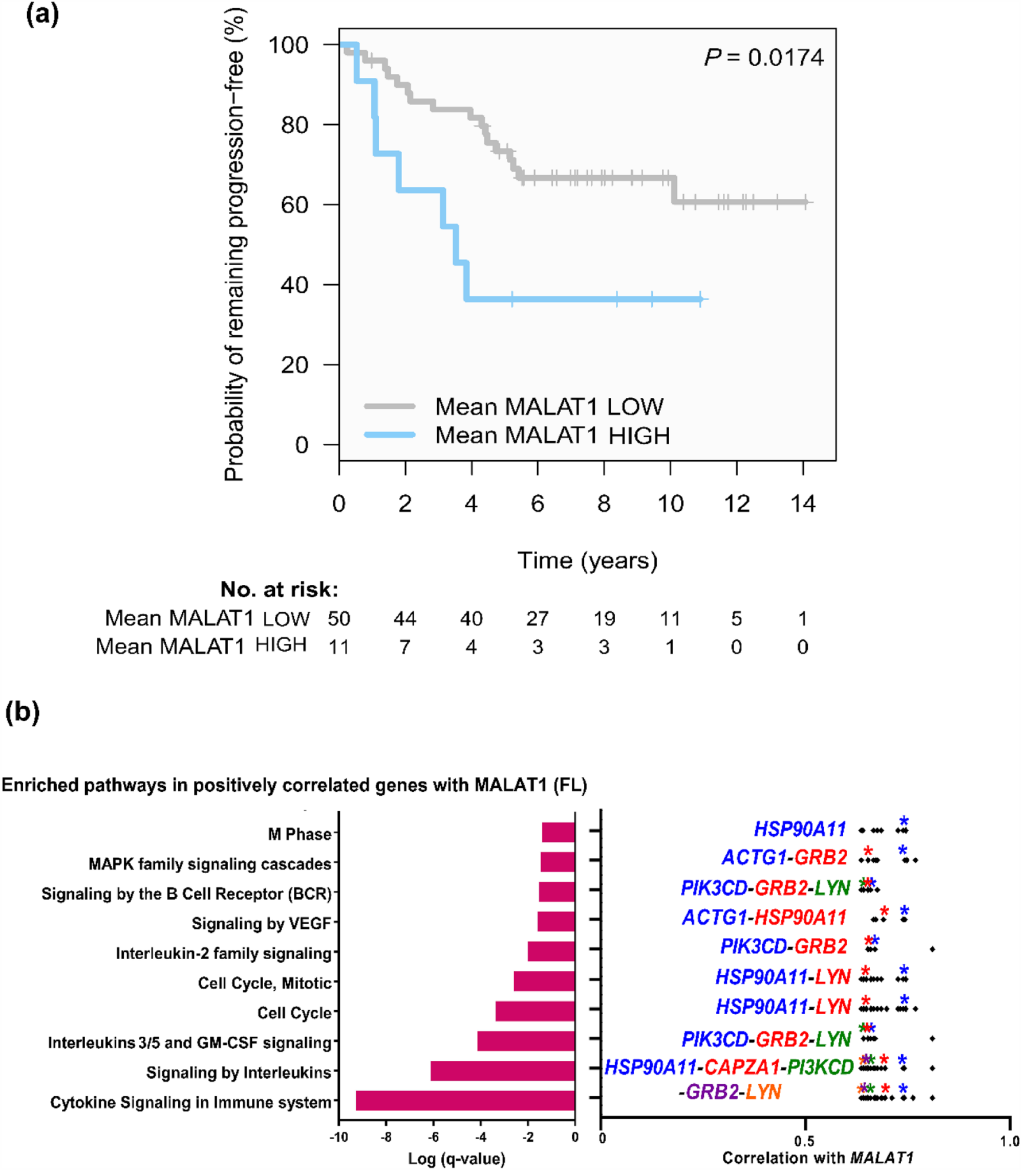
*MALAT1* expression is associated with a more aggressive behavior and is related to pathogenetic pathways in FL. (**a**) Kaplan–Meier curves for progression-free survival (PFS) according to *MALAT1* expression in FL**.** Patients with high *MALAT1* expression had a significantly shorter PFS than those with low levels. (**b**) Summary of most relevant significant pathway enrichments found with Metascape tool among coding genes positively correlated with *MALAT1* in FL samples (left panel). Only statistically significant Reactome pathways after multiple comparison correction are shown for compact representation. Correlation values of the core enriched genes of the corresponding pathways are depicted in the right panel with relevant genes marked in colored asterisks according to the degree of correlation (in blue those more highly correlated with *MALAT1*).

Based on the results of *MALAT1* in CLL and its potential association with the LN microenvironment, we further explored the role of *MALAT1* in FL, another indolent B cell lymphoid neoplasm with predominant nodal presentation. *MALAT1* expression levels were analyzed by qRT-PCR in a series of 61 grade 1-3A FL (FL#1 series, Table 1). These patients had been homogenously treated with R-CHOP. Using the *maxstat* algorithm, we identified two groups of FL with high (N = 11) and low (N = 50) *MALAT1* expression. FL cases with high *MALAT1* expression had a significantly shorter progression-free survival (PFS) than cases with low expression (*P* = 0.017) (Fig. 5a). However, no significant differences were observed in transformation to DLBCL or OS between cases with low and high expression (*P* = 0.088 and *P* = 0.0177) (Supplementary Fig. S9 online). Other clinical, biological, and histological characteristics were similar in FL with high and low *MALAT1* expression, including parameters of known prognostic impact in FL such as high-risk FLIPI, stage or histological grade (Supplementary Table S10 online), suggesting that the prognostic value of *MALAT1* is independent of these parameters.

To explore the possible biological role of *MALAT1* expression in FL we reanalyzed microarray expression data previously generated on purified FL B-cells^40^. We observed that the coding genes positively correlating with *MALAT1* expression (Supplementary Table S11 online) were associated with cell proliferation and other pathways like VEGF, MAPK, interleukin signaling (including some particular pathways related to IL-3 and IL-5) and BCR signaling, also described as involved in FL oncogenesis (Fig. 5b and Supplementary Table S12 online)^41,42^. Moreover, among the core genes positively correlated with *MALAT1* we noticed several genes which function is concordant with the aggressiveness of high *MALAT1* levels observed in FL (Fig. 5b and Supplementary Table S13 online). In this way, *PIK3CD, LYN* and *GRB2* were involved in several pathways and *PIK3CD* was previously described as predictor of poor prognosis in FL^43^. We also found others genes related to cytokine and interleukin signaling and other pathways involved in tumor progression (Fig. 5b)^44–49^.

In a subsequent analysis of differentially expressed genes between FL with high and low *MALAT1* expression in FL#2 cohort we identified 26 genes (Supplementary Fig. S10 and Table S14 online). The top overexpressed gene was *IL-7* which high expression has been previously related to the activation of Tfh cells in FL^50^. The second overexpressed gene was *GNG11* previously found overexpressed in FL with shorter response to R-CHOP therapy^51^. The GSEA analysis on the global gene list ranked by differential expression did not show relevant pathways related to FL.

Finally, we performed a comparison of all genes involved in pathways significantly enriched in FL and compared to those found in CLL (considering together U-CLL and M-CLL gene lists). A large proportion of those genes were exclusive for each lymphoma type (88% in FL and 99% in CLL) (Supplementary Fig. S11 and Table S15 online). Concordantly, cell cycle-related or MAPK pathways that were initially observed in both neoplasms involved different sets of *MALAT1* positively correlated genes (Supplementary Tables S16 and S17 online). On the other hand, a total of 12 genes were found in common in the comparative analysis, including several involved in cytokine and interleukin signaling (*CAPZA1*), cell cycle (*GAR1*), or both (*HSP90AA1*, *LMNB1* and *LYN*)(Supplementary Table S18 online).

## Discussion

In this report, we provide novel insights into the clinical and biological role of *MALAT1* in indolent B cell neoplasms. Our study revealed that *MALAT1* upregulation was associated with a detrimental clinical behavior in the different entities studied, associated with a shorter TTT in CLL or shorter PFS in FL. Interestingly, *MALAT1* upregulation was a prognostic factor in CLL independently of the IGHV mutational status, epigenetic subgroups or Binet stage. In FL, *MALAT1* overexpression was also associated with shorter progression-free survival although the clinical and biological features of patients with low and high expression were similar.

We also studied the potential causes and consequences of *MALAT1* upregulation that might justify its clinical behavior in indolent B cell neoplasms. In CLL, *MALAT1* mutations were rare and not related to the expression of the gene. This finding is similar to those in other neoplasms such as bladder cancer, head and neck squamous cell cancer, and lung adenocarcinomas, in which single nucleotides variants and indels were found, although considered passenger events caused by a transcription-associated mutational process^52,53^. Moreover, we also considered if changes in the methylation status of *MALAT1* could be associated with its upregulation as previously showed in lung cancer^54^. However, the region studied in CLL was found mainly hypomethylated and without any clear association with *MALAT1* expression differences across patients.

Therefore, other mechanisms should be related to *MALAT1* overexpression. In this sense, the transcription factors *HIF1α* and *STAT3* induced by microenvironmental factors such as hypoxia and cytokines/interleukins bind to *MALAT1* promoter increasing its expression^55–58^. In CLL, microenvironment stimulation occurs in the LN and is a key process for activation, proliferation and survival of these tumoral B cells^59^. Interestingly, our guilt-by-association analysis on the coding genes correlated with *MALAT1* in CLL as well as the differentially expressed genes in cases with high and low MALAT1 revealed significant enrichment in genes signature highly expressed in nodal CLL^17^, and interleukin/cytokine-signalling^27^, suggesting a close relationship between *MALAT1* expression and the influence of the nodal microenvironment. Interestingly, we found that *MALAT1* levels remained similar in PB compared to LN/BM paired CLL samples, supporting that the clinical value of *MALAT1* expression found in PB is mirroring expression differences in other compartments as in the LN, and that could be associated with the degree of the microenvironment stimulation. Other pathways upregulated in relation to *MALAT1* overexpression were mainly involved in cell cycle regulation and signal transduction, also known to be stimulated by nodal microenvironment and related to CLL aggressiveness^22,60^.

Intriguingly, we observed *MALAT1* downregulation in the aggressive RT compared to their paired CLL phase. This result could be related to the increased genomic alterations acquired in RT that may drive proliferation and survival of tumor cells more independently of the microenvironment^38,61^. Interestingly, one of the pathways downregulated in association with high *MALAT1* expression in CLL was OXPHOS. These findings are concordant with the high OXPHOS activity recently identified as characteristic of RT together with downregulation of the BCR signaling as additional evidence of increasing independence of transformed tumor cells from the microenvironment stimuli^38^.

*MALAT1* expression in CLL was not related to any of the major genomic alteration with known prognostic impact in the evolution of the disease. This finding is concordant with the predictive value of *MALAT1* overexpression for shorter TTT together with the mutational status of the IGHV and Binet stage, independently of strong driver alterations, including *TP53* mutations or deletions (see Supplementary Fig. 3b,c online). However, the pathway analysis of the genes correlated to *MALAT1* expression showed that one of the significant altered pathways was transcriptional regulation of *TP53* (see Fig. 3b). One of the genes downregulated in association with *MALAT1* was *PIN1* that seems to be required for the transcriptional activity of wild-type *TP53*^37^, suggesting that the low levels of *PIN1* found in CLL with high *MALAT1* expression could contribute to impair *TP53* activity even in absence of alterations in this tumor suppressor gene.

In FL, our study also revealed biological insights that seem to explain the clinical behavior of *MALAT1* upregulation in these neoplasms and its relationship to the aggressiveness of the tumors. Coding genes correlated with *MALAT1* expression in FL were enriched in cell cycle-related processes as well as pathways related to cell proliferation, migration and angiogenesis, such as MAPK and VEGF pathways, which have been previously linked to *MALAT1* function in other tumor models^62^. We also found that high *MALAT1* expression was significantly associated with high levels of genes involved in several other pathways as BCR and interleukin signaling, such as *LYN* and *PIK3CD*, the expression of which promote cell growth and have been associated with poor prognosis in FL^43,63^. Moreover, the study of differentially expressed genes between case with high and low *MALAT1* identified *IL-7* previously described as upregulated in FL B-cells paralleling the levels of activation of Tfh cells^50^ and *GNG11* highly expressed in FL with short response to R-CHOP therapy^51^. All these are relevant associations supporting the clinical impact of *MALAT1* in FL even though our series included a relative limited number of cases.

Finally, the comparison of *MALAT1* associated pathways and the involved genes showed a low degree of overlapping in the corresponding gene signatures, even in cell cycle-related or MAPK pathways that were initially observed as enriched in both neoplasms. These results suggest that *MALAT1* could be contributing to the regulation of different transcriptional gene sets in both lymphomas. In spite of these differences, we also observed that processes as cell proliferation and MAPK pathways were potentially commonly affected by *MALAT1* upregulation, and thus could explain its common clinical impact in both neoplasms.

In summary, these findings highlight that *MALAT1* overexpression plays a role in the pathobiology and clinical behavior of indolent B cell neoplasms related to a more aggressive behavior of the tumors with higher *MALAT1* levels. Particularly in CLL, *MALAT1* could serve as a clinical biomarker that seems to be a surrogate indicator of the degree of stimuli the CLL cells receive from the microenvironment. Therefore, *MALAT1* expression could be taken in account in further studies as complementary to other known prognostic factors to improve the clinical management of these patients.

## Material and methods

### Description of the transcriptional data and patient cohorts/features

This study exploited previously published genome wide transcriptional data from CLL and FL. Regarding CLL, we reanalyzed our RNA-seq data from PB pre-treatment samples obtained from 266 patients, and 25 additional monoclonal B cell lymphocytosis donors (MBL) of the Spanish ICGC consortium with full clinical annotations^13^ (MBL and CLL#1 series, Table 1). The CLL subtypes according to IGHV mutational status and epigenetic subtypes were identified following standard methods published elsewhere^64,65^. *MALAT1* methylation data was obtained from a previous work that also included the patients of CLL#1, using 450k array^16^. The studied region spanned the genomic coordinates, chr11:65265233–65273940 (hg19) and included 9 CpGs (one in the CpG island, three at the shelf and 5 at the shore) located along the gene body. We also compared the *MALAT1* expression in 6 paired samples of CLL and Richter transformation from RNAseq data already published (CLL#2 series, Table 1)^38^. Finally, we have also analyzed the microarray data from the GSE21029 GEO dataset (CLL#3 series, Table 1), including 17 patients with paired PB/LN samples, and 19 patients with paired PB/BM.

For the analysis of *MALAT1* in FL, we extracted RNA from formalin-fixed paraffin-embedded (FFPE) LN samples of 61 FL patients (stages 1 to 3A) from the Hematopathology collection of the Biobank of the Hospital Clínic de Barcelona-IDIBAPS (Spain) (FL#1 series, Table 1). In addition, microarray data generated from sorted FL neoplastic cells of 23 patients were obtained from the GSE107367 GEO dataset^40^ (FL#2 series, Table 1).

### RNA extraction and qRT-PCR

For the 61 FFPE FL samples, 3–10 cuts of 10 µm each were used per sample for RNA extraction using Allprep DNA/RNA FFPE kit (Qiagen). RNA integrity was analyzed with TS4200 using DV200 index as recommended by the manufacturer (Agilent). In this way, samples with DV200 less than 35% were excluded to avoid excessively degraded material. Reverse transcriptase reaction was performed using High-Capacity Reverse transcription kit (Applied Biosystems) with an RNA input of 90 ng.

*MALAT1* expression was analyzed by qRT-PCR using a short amplicon specifically designed for FFPE samples. *MALAT1* expression levels were normalized using three reference genes (*ACTB*, *GAPDH* and *YWHAZ*) which have been previously used in B-cell lymphomas, including FL samples^66,67^. Primer quantities were optimized for each amplicon to reach a high efficiency (range: 90–96%) and their sequences were: 5'-CCCCTTCCCTAGGGGATTTCA-3' (*MALAT1* forward), 5'- AAGCCCACAGGAACAAGTCC-3' (*MALAT1* reverse), 5'- CCAACCGCGAGAAGATGAC-3'(ACTB forward), 5'-TAGCACAGCCTGGATAGCAA-3' (*ACTB* reverse), 5'- AGGTGAAGGTCGGAGTCA-3'(*GAPDH* forward), 5' CAACAATATCCACTTTACCAGAGTTAA-3' (*GAPDH* reverse), 5'-CAAAGACAGCACGCTAATAATGCA-3' (*YWHAZ* forward), and 5'-TCAGCTTCGTCTCCTTGGGTA-3' (*YWHAZ* reverse). Amplification reactions were carried out using PowerUp^Tm^ SYBR™ Green Master Mix (Applied Biosystems, Foster City, CA) following supplier's recommendations in a Step One Plus thermocycler (Applied Biosystems). Transcript expression relative quantification was performed referred to a calibrator sample of universal human reference RNA (Invitrogen).

### Bioinformatic analyses and statistics

Microarray data were normalized with RMA-based methodology and used to analyze the expression levels of *MALAT1* transcript. Although the expression microarrays were enriched in probe sets for coding genes, several probe sets that hybridize exclusively to the *MALAT1* transcript were initially included. The specificity of the probe sets was confirmed in the Affymetrix database (NettAffx™). Only one probe set was excluded (223579_s_at) at this stage. The correlations among the different probe sets were checked by strand and another probe set was excluded as an outlier (224559_at). Finally, the mean values of the remaining probe sets were retained as reliable for measuring the expression levels of *MALAT1* (probe sets 1558678_s_at, 223940_x_at, 224558_s_at, 224567_x_at, 224568_x_at and 226675_s_at).

In the clinical association studies, optimal cut-off points for *MALAT1* expression groups were obtained using the *maxstat* algorithm which optimized the log-rank statistics (*maxstat* package, R Software, Vienna). Cumulative incidence and Kaplan–Meier curves, scatterplots, and box plot graphs were generated using both the R environment and GraphPad Prism v7. Univariate and multivariate Fine-Gray regression models considering *MALAT1* as a continuous variable (as a more stringent statistical approach) were used for measuring its impact on time to treatment (TTT). Univariate analysis of *MALAT1* as a continuous variable was performed with the Cox regression model for overall survival (OS). Both calculations were performed with R v.4.0.3. Student's t-test was used to evaluate the differences in the mean expression of *MALAT1* among subtypes and molecular factors with previously described prognostic value in CLL. Paired t-test was used to evaluate differences in *MALAT1* expression between paired samples of CLL in PB and LN^39^. Linear regression on scatter plots was representing the correlation between *MALAT1* expression and the epiCMIT score (epigenetically-determined Cumulative MIToses)^16^.

To identify potential target genes, ranked lists of coding genes according to the degree of Pearson's correlation to *MALAT1* were obtained for the different indolent B-cell lymphomas under study using either RNA-seq (CLL#1) or microarray data (FL#2). Only those statistically significant correlations after correction for multiple comparisons (adjusted *p*-value < 0.05) were considered for the downstream analyses. We separately analyzed positive and negative correlations of coding genes with *MALAT1* using the webtool Metascape^68^. The Metascape output of enriched (Reactome) pathways were obtained as tables, and summary plots depicting relevant pathways with very high statistical significance (q < 0.05) were prepared with Graphpad Prism v7. Furthermore, we used Gene Set Enrichment Analysis (GSEA v3.0 from http://www.gsea-msigdb.org) to perform additional pathway enrichment analyses. First we analyzed the ranked list of coding genes correlated with *MALAT1* for M-CLL and U-CLL subtypes using the C2 curated gene signatures from MSigDB/GSEA website and several specific signatures previously related to differences between LN and PB in CLL^17^. Second, GSEA were applied to global expression matrices for each CLL subtype ranked by their degree of differential expression.

The differential expression analysis comparing samples subsets of *MALAT1* high and low expression was performed using Limma in R v.4.0.3. Those subsets were defined using the clinically significant cutoff found in the CLL#1 series, whereas for the FL#2 series the median value of *MALAT1* expression was used. The adjustment of *p*-values for multiple comparisons was performed only in CLL#1, accordingly to the limited sample size of the FL#2 studied series. MORPHEUS software platform (https://software.broadinstitute.org/morpheus) was used to generate the plots using euclidean metrics of hierarchical clusters to depict the significantly overexpressed/downregulated genes found in all these comparisons.

### Ethics approval and consent to participate

Samples from FL patients were obtained from the Hematopathology collection of the Biobank of the Hospital Clínic de Barcelona-IDIBAPS (Spain). RNAseq data and full clinical annotations from CLL patients were obtained from the Spanish ICGC consortium, which had an approval of the Institutional Review Board of Hospital Clinic de Barcelona, in accordance with national regulations and the Declaration of Helsinki. All patients provided written informed consent. The remaining data from other samples were obtained from GEO microarray public repository including previously published works with their particular ethical compliances stated in the original articles.

### Supplementary Information

Supplementary Table S1.

Supplementary Table S2.

Supplementary Table S3.

Supplementary Table S4.

Supplementary Table S5.

Supplementary Table S6.

Supplementary Table S7.

Supplementary Table S8.

Supplementary Table S9.

Supplementary Table S10.

Supplementary Table S11.

Supplementary Table S12.

Supplementary Table S13.

Supplementary Table S14.

Supplementary Table S15.

Supplementary Table S16.

Supplementary Table S17.

Supplementary Table S18.

Supplementary Figures.

Supplementary Legends.

## Data Availability

The data that support the findings of this study are available from the corresponding author upon reasonable request.
