# Supplementary material for: MALAT1 expression is associated with aggressive behavior in indolent B-cell neoplasms
Source: Sci Rep. 2023 Oct 6;13:16839. doi: 10.1038/s41598-023-44174-8 (PMC10558466; doi:10.1038/s41598-023-44174-8)

SUPPLEMENTARY FIGURES

MALAT1 **Expression is Associated with Aggressive Behavior in Indolent B-Cell Neoplasms**

Elena María Fernández-Garnacho, Ferran Nadeu, Silvia Martín, Pablo Mozas, Andrea Rivero, Julio Delgado, Eva Giné, Armando López-Guillermo, Martí Duran-Ferrer, Itziar Salaverria, Cristina López, Sílvia Beà, Santiago Demajo, Pedro Jares, Xose S Puente, José Ignacio Martín-Subero, Elías Campo, Lluís Hernández

Suppl. Fig 1

(a)

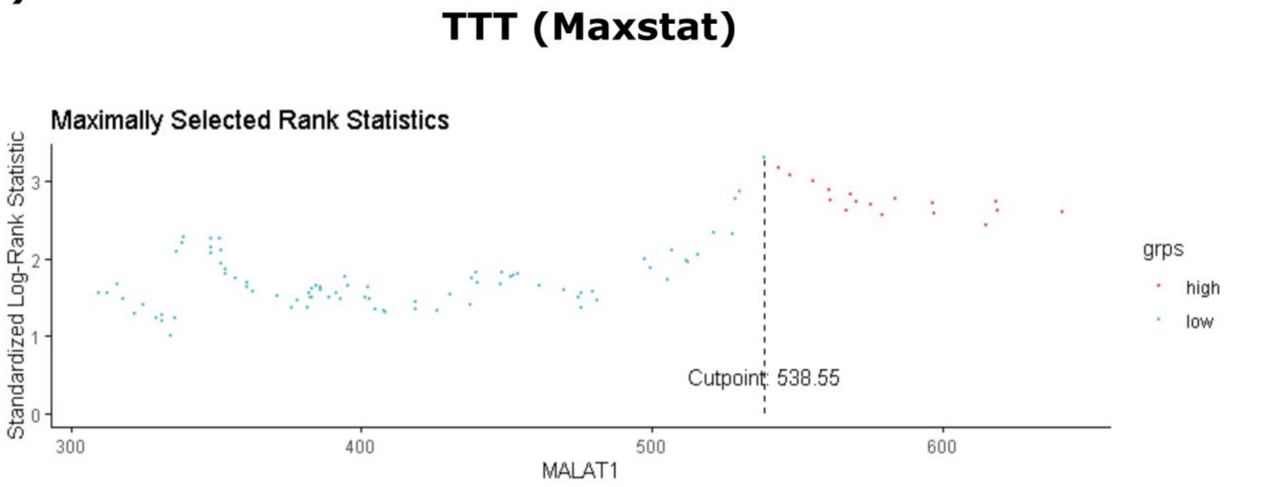

(b)

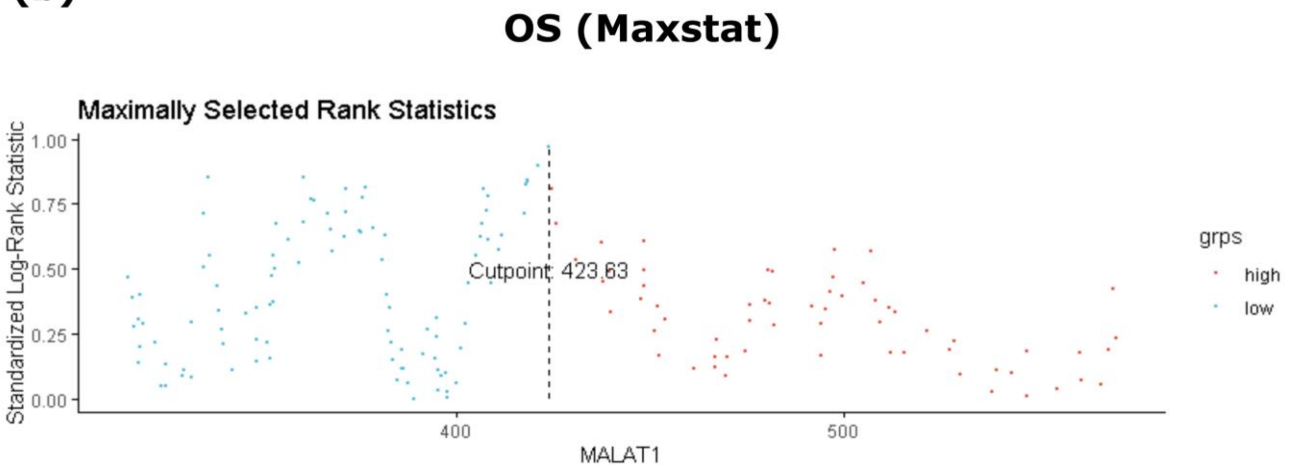

Suppl. Fig 2

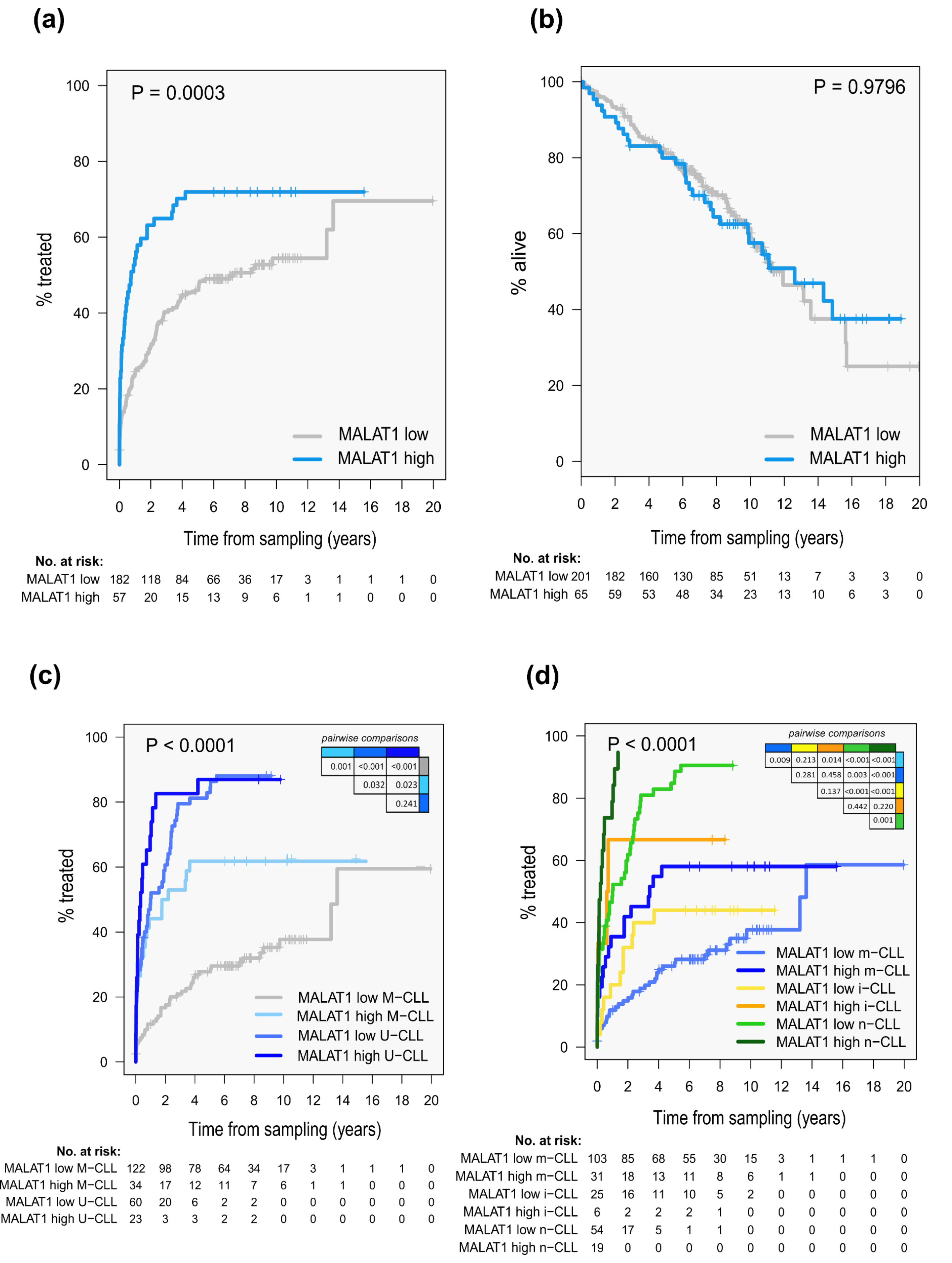
(a)
P = 0.0003
% treated
100
80
60
40
20
0
MALAT1 low
MALAT1 high
0 2 4 6 8 10 12 14 16 18 20
Time from sampling (years)
No. at risk:
MALAT1 low 182 118 84 66 36 17 3 1 1 1 0
MALAT1 high 57 20 15 13 9 6 1 1 0 0 0
(b)
P = 0.9796
% alive
100
80
60
40
20
0
MALAT1 low
MALAT1 high
0 2 4 6 8 10 12 14 16 18 20
Time from sampling (years)
No. at risk:
MALAT1 low 201 182 160 130 85 51 13 7 3 3 0
MALAT1 high 65 59 53 48 34 23 13 10 6 3 0
(c)
P < 0.0001
pairwise comparisons
0.001 <0.001 <0.001
0.032 0.023
0.241
% treated
100
80
60
40
20
0
MALAT1 low M−CLL
MALAT1 high M−CLL
MALAT1 low U−CLL
MALAT1 high U−CLL
0 2 4 6 8 10 12 14 16 18 20
Time from sampling (years)
No. at risk:
MALAT1 low M−CLL 122 98 78 64 34 17 3 1 1 1 0
MALAT1 high M−CLL 34 17 12 11 7 6 1 1 0 0 0
MALAT1 low U−CLL 60 20 6 2 2 0 0 0 0 0 0
MALAT1 high U−CLL 23 3 3 2 2 0 0 0 0 0 0
(d)
P < 0.0001
pairwise comparisons
0.009 0.213 0.014 <0.001 <0.001
0.281 0.458 0.003 <0.001
0.137 <0.001 <0.001
0.442 0.220
0.001
% treated
100
80
60
40
20
0
MALAT1 low m−CLL
MALAT1 high m−CLL
MALAT1 low i−CLL
MALAT1 high i−CLL
MALAT1 low n−CLL
MALAT1 high n−CLL
0 2 4 6 8 10 12 14 16 18 20
Time from sampling (years)
No. at risk:
MALAT1 low m−CLL 103 85 68 55 30 15 3 1 1 1 0
MALAT1 high m−CLL 31 18 13 11 8 6 1 1 0 0 0
MALAT1 low i−CLL 25 16 11 10 5 2 0 0 0 0 0
MALAT1 high i−CLL 6 2 2 2 1 0 0 0 0 0 0
MALAT1 low n−CLL 54 17 5 1 1 0 0 0 0 0 0
MALAT1 high n−CLL 19 0 0 0 0 0 0 0 0 0 0

Suppl. Fig 3

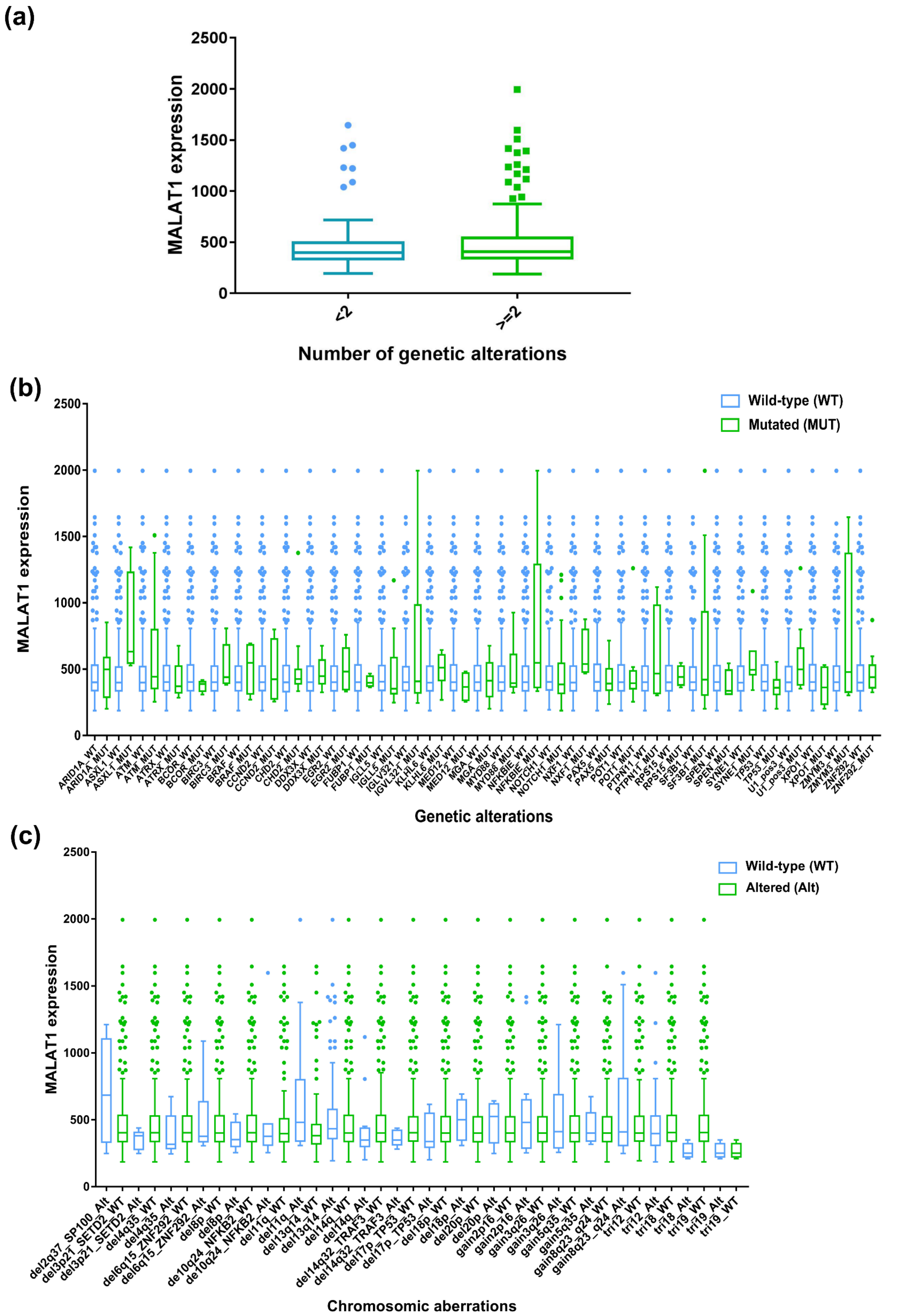

(a)
MALAT1 expression
2500
2000
1500
1000
500
0
<2
>=2
Number of genetic alterations
(b)
Wild-type (WT)
Mutated (MUT)
MALAT1 expression
Genetic alterations
(c)
Wild-type (WT)
Altered (Alt)
MALAT1 expression
Chromosomic aberrations

Suppl. Fig 4

(a)

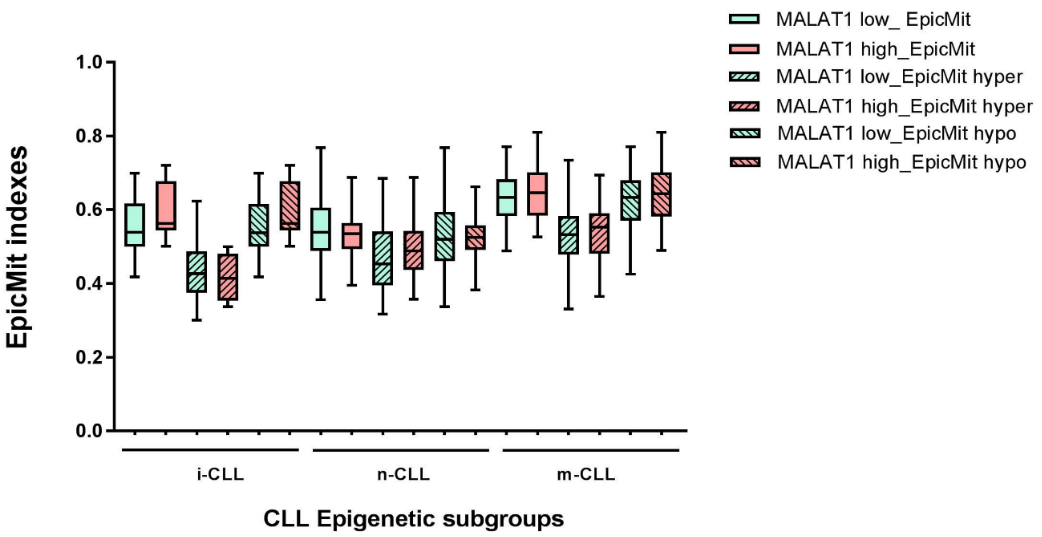

(b)

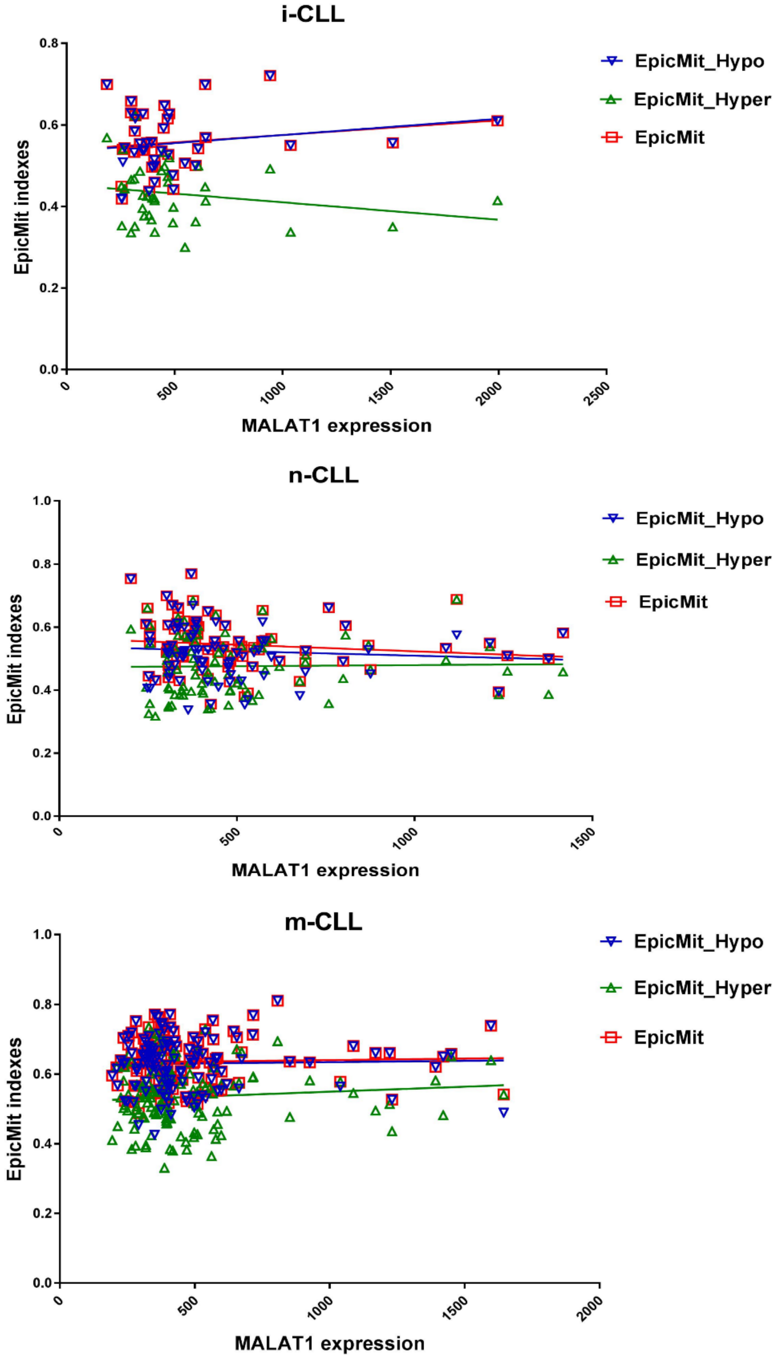

Suppl. Fig 5

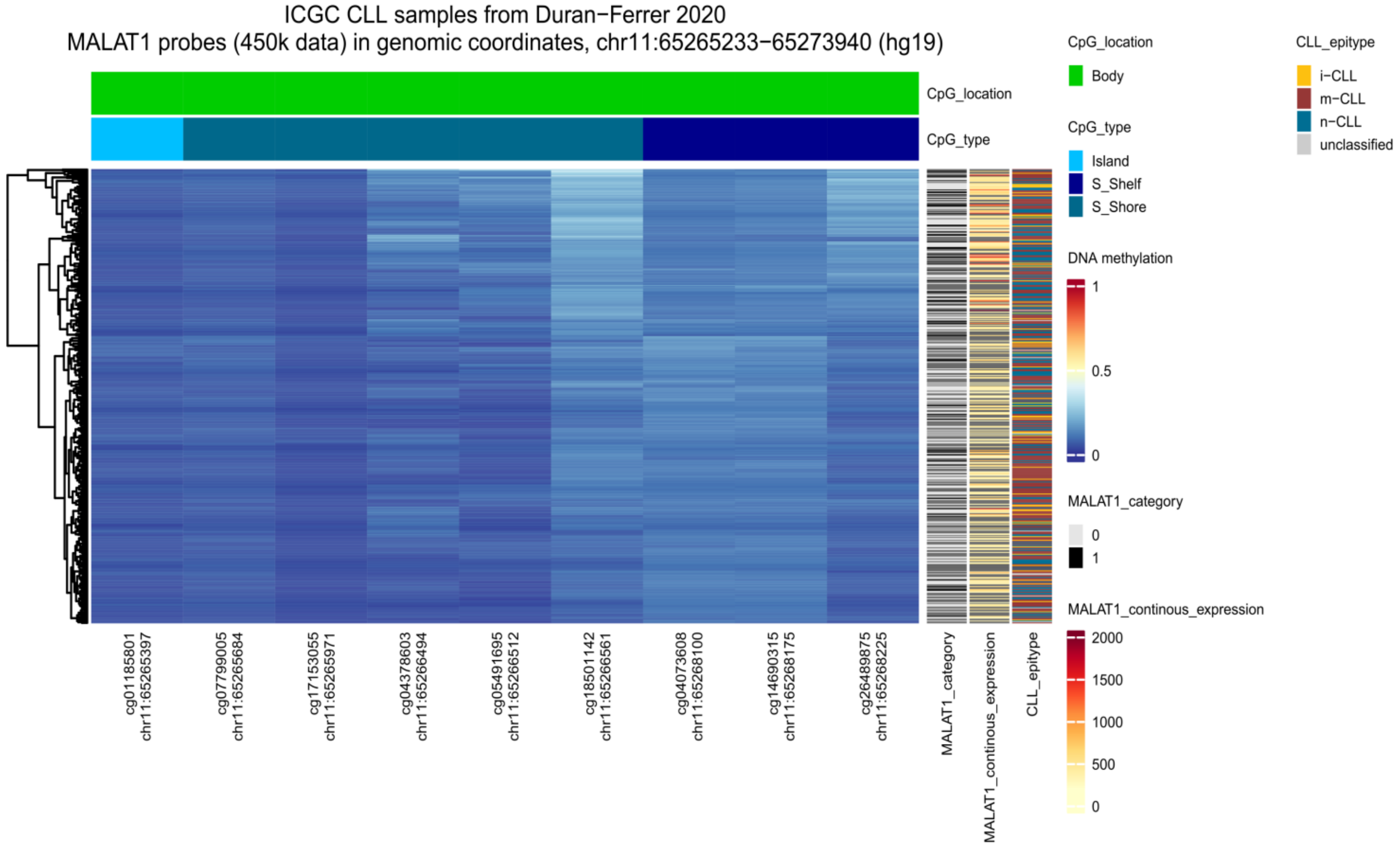
ICGC CLL samples from Duran−Ferrer 2020
MALAT1 probes (450k data) in genomic coordinates, chr11:65265233−65273940 (hg19)
CpG_location
CpG_type
cg01185801 chr11:65265397
cg07799005 chr11:65265684
cg17153055 chr11:65265971
cg04378603 chr11:65266494
cg05491695 chr11:65266512
cg18501142 chr11:65266561
cg04073608 chr11:65268100
cg14690315 chr11:65268175
cg26489875 chr11:65268225
MALAT1_category
MALAT1_continous_expression
CLL_epitype
CpG_location
Body
CpG_type
Island
S_Shelf
S_Shore
DNA methylation
1
0.5
0
MALAT1_category
0
1
MALAT1_continous_expression
2000
1500
1000
500
0
CLL_epitype
i−CLL
m−CLL
n−CLL
unclassified

Suppl. Fig 6

(a)

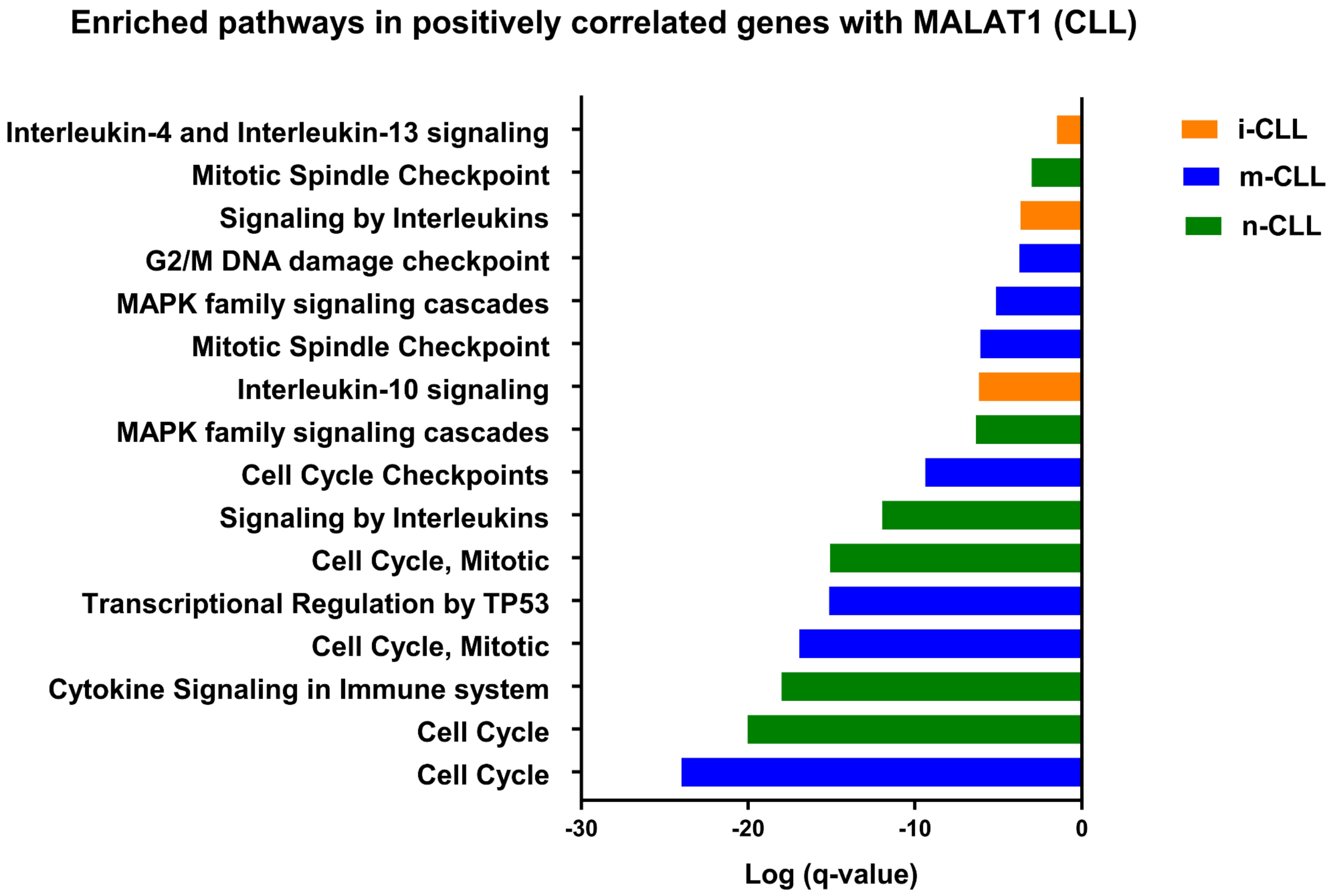

(b)

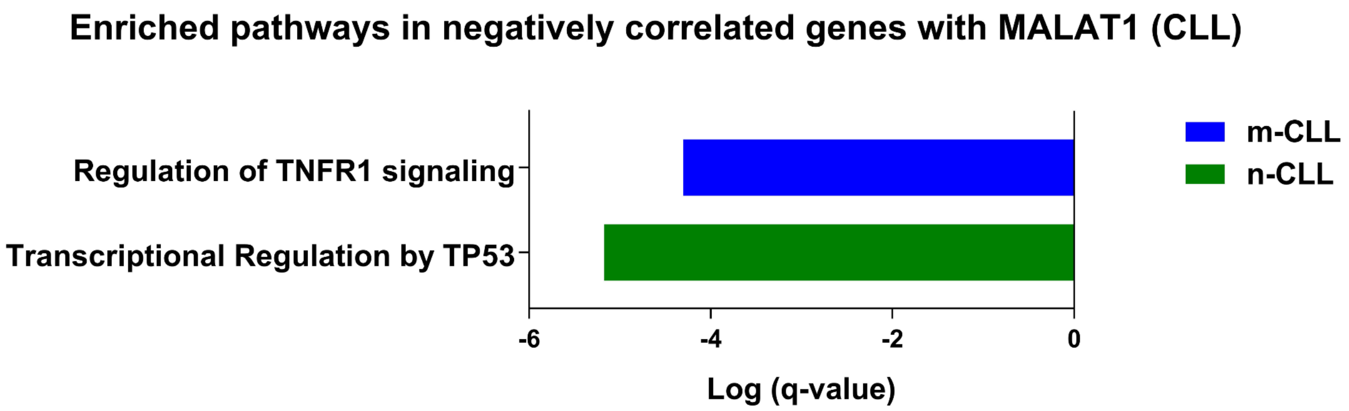

Suppl. Fig 7

U-CLL

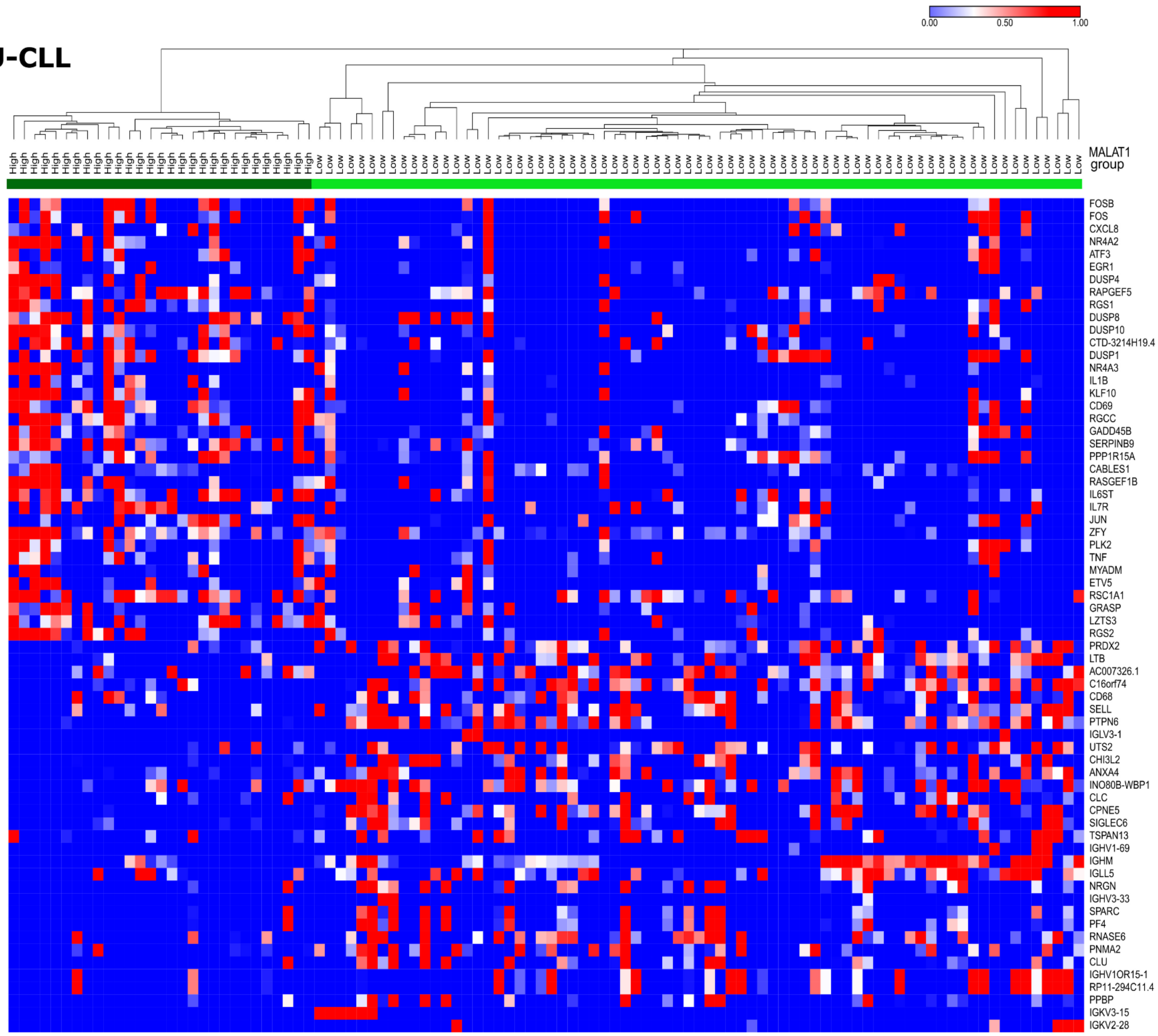

M-CLL

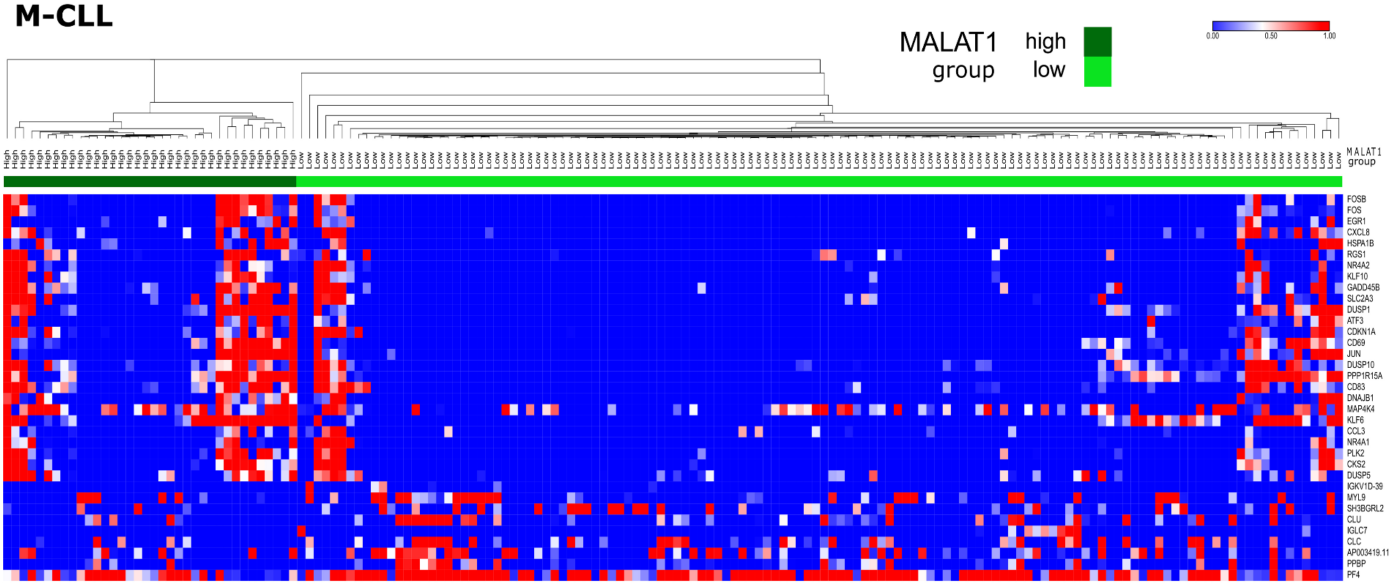

Suppl. Fig 8

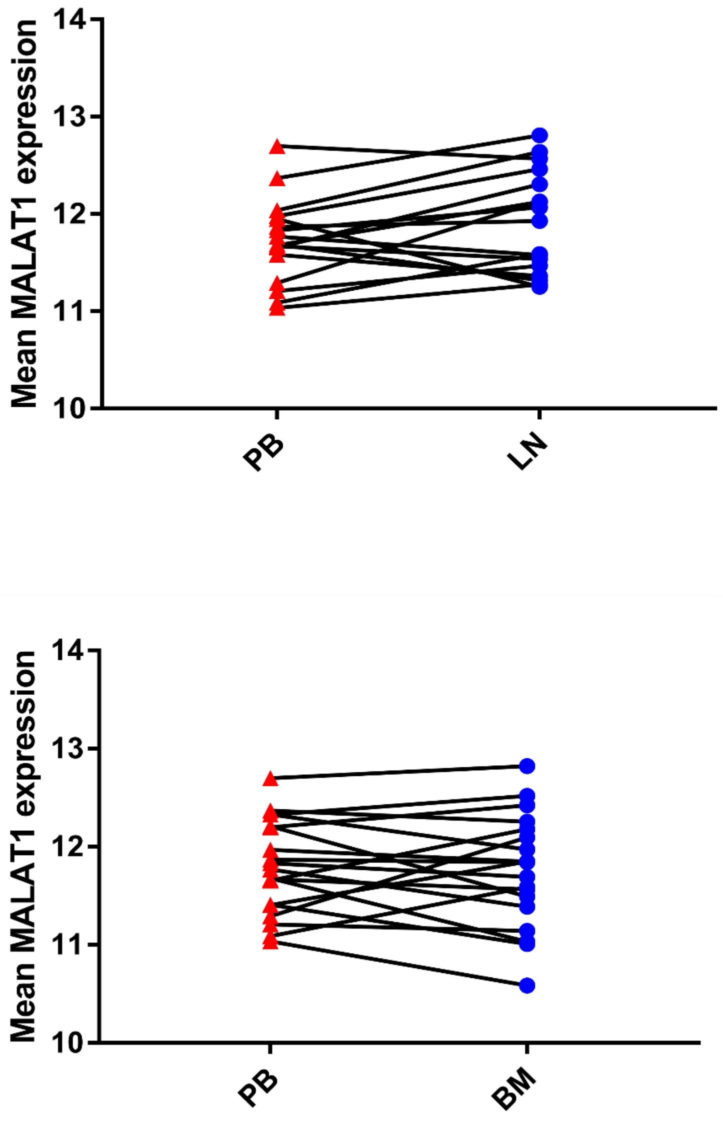

Mean MALAT1 expression
14
13
12
11
10
PB
LN
Mean MALAT1 expression
14
13
12
11
10
PB
BM

Suppl. Fig 9

(a)

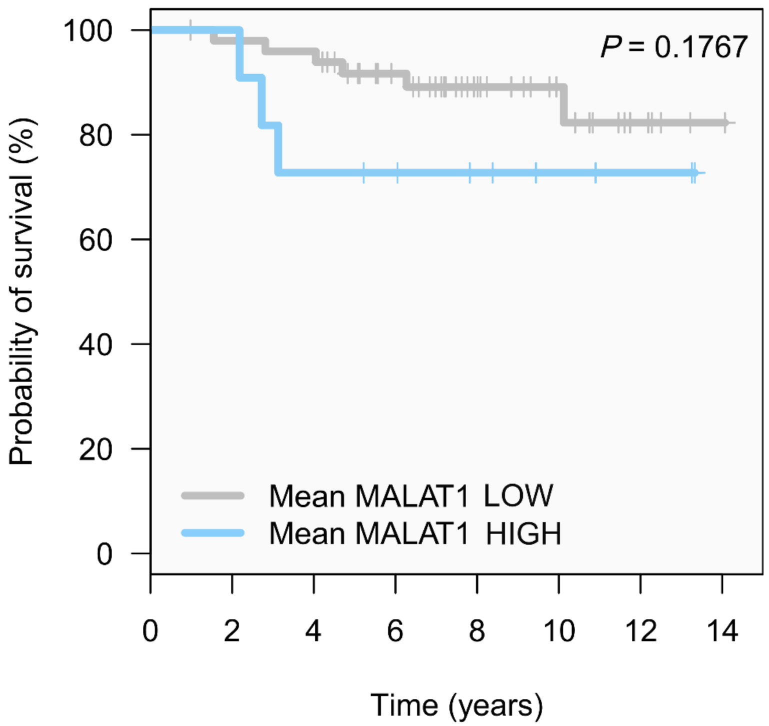

No. at risk:

Mean MALAT1 LOW	50	48	47	36	23	13	5	1
Mean MALAT1 HIGH	11	11	8	7	5	3	2	0

(b)

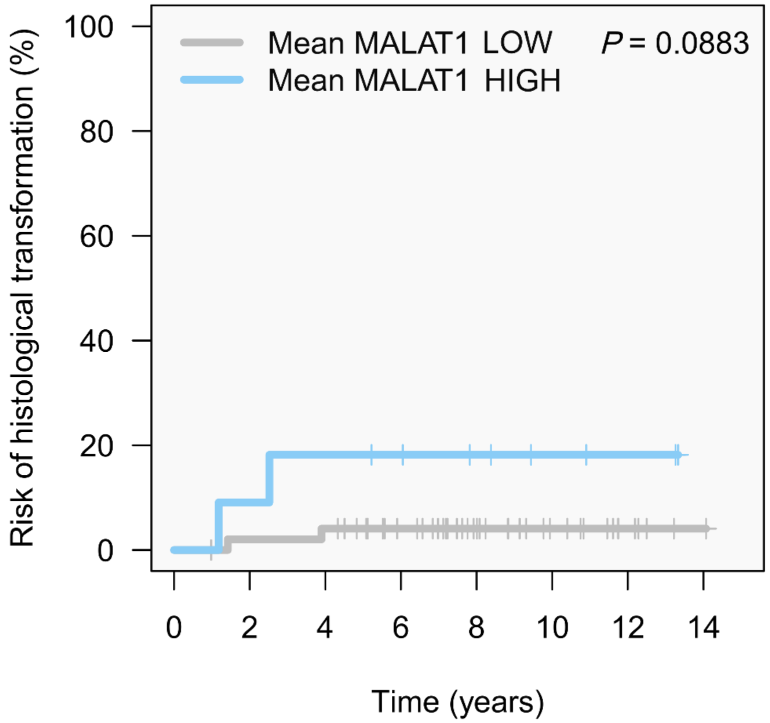

No. at risk:

Mean MALAT1 LOW	50	47	45	36	23	13	5	1
Mean MALAT1 HIGH	11	10	8	7	5	3	2	0

Suppl. Fig 10

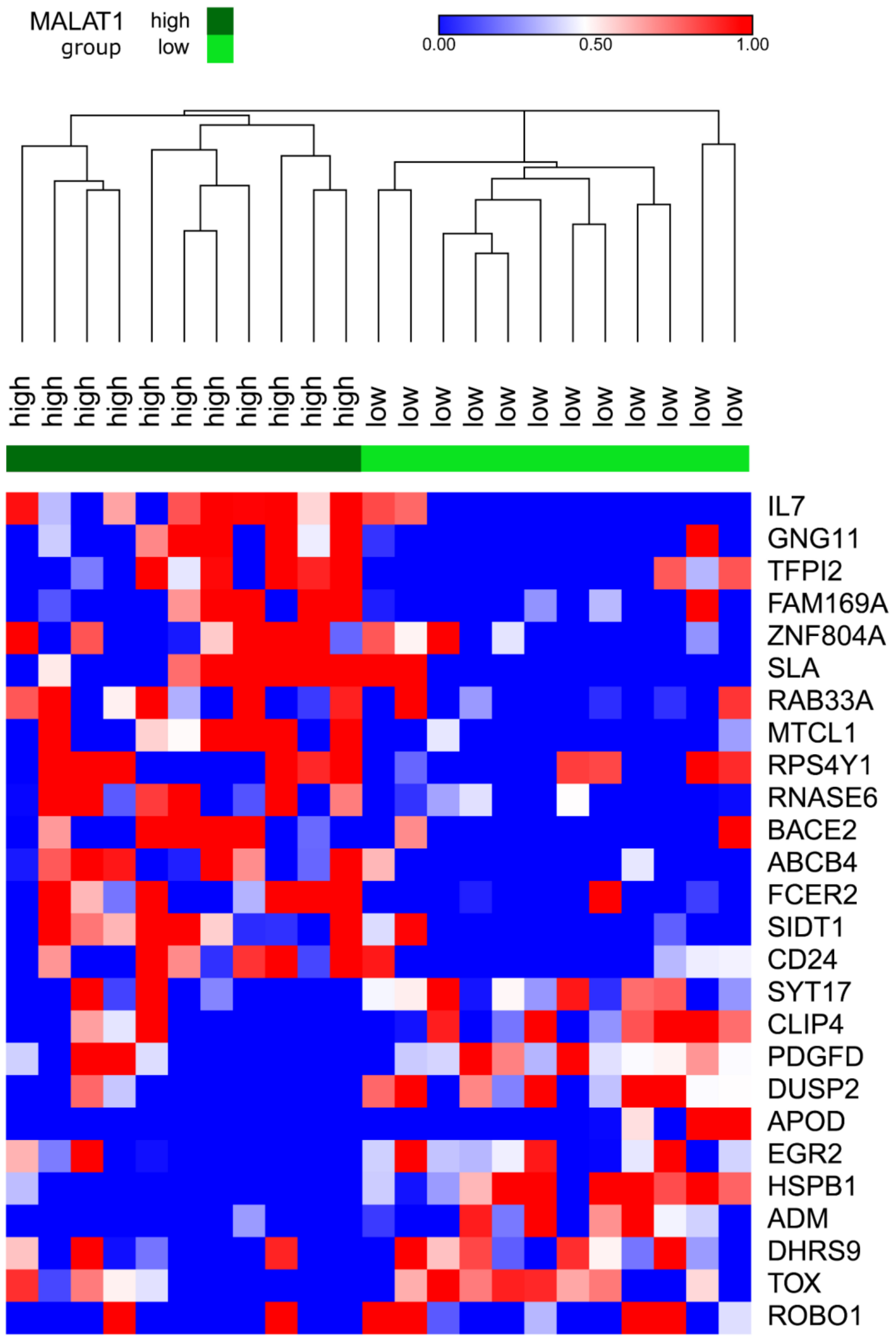

MALAT1 group
high
low
0.00
0.50
1.00
high high high high high high high high high high high low low low low low low low low low low low low
IL7
GNG11
TFPI2
FAM169A
ZNF804A
SLA
RAB33A
MTCL1
RPS4Y1
RNASE6
BACE2
ABCB4
FCER2
SIDT1
CD24
SYT17
CLIP4
PDGFD
DUSP2
APOD
EGR2
HSPB1
ADM
DHRS9
TOX
ROBO1

Suppl. Fig 11

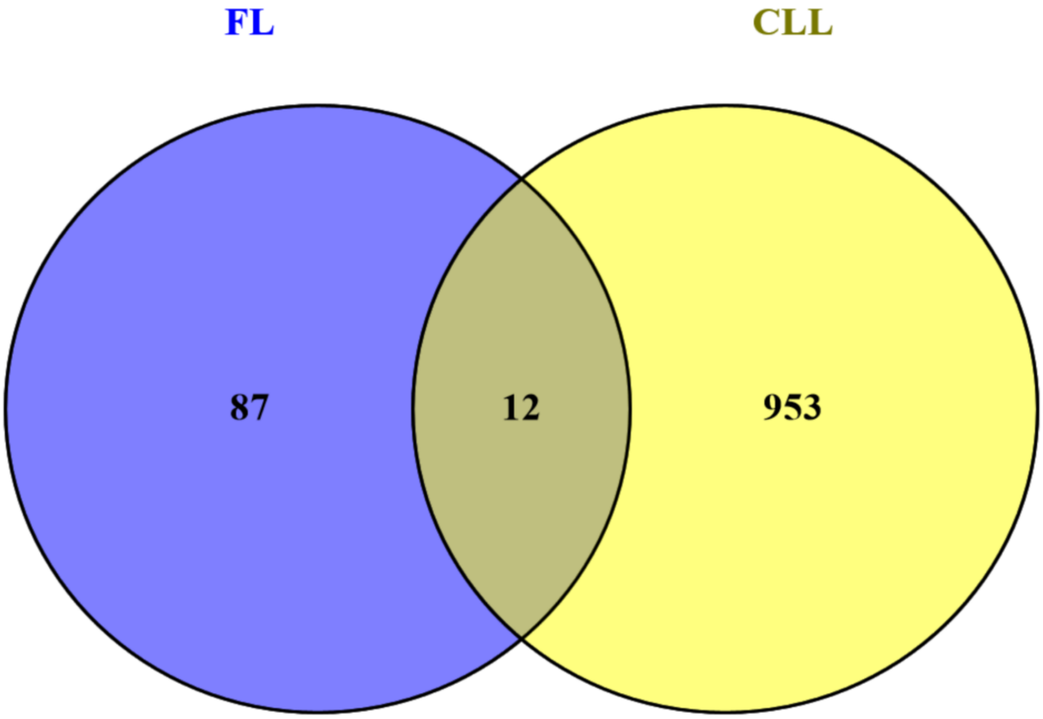

Supplement: Supplementary file 19 — Supplementary Figures. [file 41598_2023_44174_MOESM19_ESM.pdf]
