# Supplementary material for: MALAT1 expression is associated with aggressive behavior in indolent B-cell neoplasms
Source: Sci Rep. 2023 Oct 6;13:16839. doi: 10.1038/s41598-023-44174-8 (PMC10558466; doi:10.1038/s41598-023-44174-8)
Supplement: Supplementary file 20 — Supplementary Legends. [file 41598_2023_44174_MOESM20_ESM.docx]

**Supplementary Figure Legends**

**Suppl. Fig. 1. (a)** Optimal cut-off established by *maxstat* algorithm regarding *MALAT1* expression and TTT in CLL#1. **(b)** Optimal cut-off established by *maxstat* algorithm regarding *MALAT1* expression and OS in CLL#1.

**Suppl. Fig. 2.** **(a)** Binet A CLL#1 cases with high *MALAT1* expression had a significantly shorter TTT than those with low levels. **(b)** No difference in OS was detected in all CLL cases with high or low *MALAT1* expression neither using the same cutoff defining significant differences for TTT (see Suppl. Fig1a) nor the optimal for OS (data not shown). **(c)** TTT and *MALAT1* expression levels in IGHV-mutated and unmutated Binet A CLL#1 cases. **(d)** TTT and *MALAT1* expression levels in Binet A CLL#1 epigenetic subtypes.

**Suppl. Fig. 3. (a)** Similar *MALAT1* levels were found in CLL#1 cases with a different number of genetic alterations. **(b)** No significant differences in *MALAT1* expression could be observed comparing CLL cases with (mutated) or without (wild-type) individual gene alterations after correction for multiple comparisons. **(c)** No significant differences were detected in *MALAT1* expression comparing CLL#1 cases with (altered) or without (wild-type) individual chromosomal alterations after correction for multiple comparisons.

**Suppl. Fig. 4. (a)** Boxplot showing the distribution of the values of the three epiCMIT indexes in the three epigenetic groups of CLL#1 cases analyzed according to the *MALAT1* expression categories. No significant differences were found in any comparison. **(b)** Scatterplots showing the linear correlation analyses between the three epiCMIT indexes and *MALAT1* expression values, in the three CLL epigenetic subtypes. None of these correlations were statistically significant.

**Suppl. Fig. 5.** Clustering analysis of methylation status in 9 CpGs (one in the CpG island, three at the shelf and 5 at the shore) located along the gene body of *MALAT1* using previously published DNA methylation data. No association could be observed with the *MALAT1* expression, neither considered as a continuous nor as categorical variable even within the different CLL#1 subtypes. DNA methylation values were plotted using R ComplexHeatmap package in R v.4.3.

**Suppl. Fig. 6.** Summary of most relevant significant pathway enrichments found using Metascape tool involving signatures previously related to CLL pathogenesis and poor prognosis. Separated analyses were performed involving coding genes positively (top panel) and negatively (bottom panel) correlated with *MALAT1* expression in the different epigenetic CLL#1 subtypes. Only statistically significant Reactome pathways after multiple comparison correction are shown for compact representation.

**Suppl. Fig. 7.** Heat maps of hierarchical cluster analysis of differentially expressed genes found between *MALAT1* expression groups in CLL#1 subsets defined by IGHV mutational status. Plots were performed using MORPHEUS software platform (https://software.broadinstitute.org/morpheus).

**Suppl. Fig. 8.** No significant differences in *MALAT1* expression between paired PB and LN or PB and bone marrow (BM) CLL samples from CLL#3 were observed.

**Suppl. Fig. 9. (a)** Kaplan-Meier curve showing the lack of significant differences in OS of FL#1 cases regarding *MALAT1* expression. **(b)** Cumulative incidence curves showing the lack of significant differences regarding *MALAT1* expression and the risk of transformation in FL#1 cases.

**Suppl. Fig. 10.** Heat map of the hierarchical cluster analysis of differentially expressed genes found between *MALAT1* expression groups in FL#2 subsets defined by median expression of this lncRNA. Plots were performed using MORPHEUS software platform (https://software.broadinstitute.org/morpheus).

**Suppl. Fig. 11.** Venn diagram showing the overlapping between FL#2 and CLL#1 of *MALAT1*-correlated genes in pathways found significantly enriched in separated analysis.
